# Complete Genome Sequence of a Low-Virulence Tick-Borne Encephalitis Virus Strain

**DOI:** 10.1128/genomeA.01145-16

**Published:** 2016-10-20

**Authors:** G. Dobler, M. Bestehorn, M. Antwerpen, A. Överby-Wernstedt

**Affiliations:** a Bundeswehr Institute of Microbiology, DZIF partner site Munich, Munich, Germany; b Institute for Clinical Microbiology, Umea University, Umea, Sweden

## Abstract

We report here the complete genome sequence (GenBank accession no. KX268728) of tick-borne encephalitis strain HB171/11, isolated from an *Ixodes ricinus* tick from a natural focus where human neurological disease is rare. The strain shows unique characteristics in neuroinvasiveness and neurovirulence.

## GENOME ANNOUNCEMENT

Tick-borne encephalitis virus (TBEV), a member of the genus *Flavivirus* in the family *Flaviviridae*, is the most important tick-borne virus in Europe and Asia. The virus genome consists of a single-stranded 11-kb (+ [positve sense]) RNA coding for one large polyprotein, yielding three structural proteins (C, prM, and E) and seven nonstructural proteins (NS1, NS2a, NS2b, NS3, NS4a, NS4b, and NS5). TBEV strains differ in their neuropathogenicity. We present the whole genome of a unique low-pathogenic TBEV strain, adding more data on the neuropathogenicity of TBEV strains.

The clinical symptoms of the infection by TBEV strains may range from subclinical to severe and fatal forms. The basis of this variability of clinical symptoms is unknown. However, there is some evidence that beside individual patient characteristics (e.g., age of patient and underlying disease), viral characteristics also may determine the outcome of the clinical form of infection. For the pathogenicity of a neurotropic virus, its ability to invade the central nervous system (CNS) from the peripheral route of infection (neuroinvasiveness) and its ability to cause pathological changes in the CNS (neurovirulence) in laboratory mice can be distinguished (1). In nature, TBEV strains with higher (strain Hypr) or lower (strain Neudörfl) neuropathogenicity have been described (2). However, in these strains, the neuropathogenicity could still not well be correlated with defined nucleotides in the genome. So far, only one naturally occurring strain of TBEV from Czech Republic, strain T263, is known to exhibit reduced neuroinvasiveness with reduced neuropathogenicity by the peripheral route of infection but with high neuropathogenicity when injected directly into the brain of mice (3). This reduced neuroinvasiveness could be traced to mutations in the NS2b and/or NS3 gene. We present the whole genome of a naturally occurring TBEV strain with decreased neuropathogenicity based on a decreased neuroinvasiveness and neurovirulence.

TBEV strain MucAr HB171/11 was isolated from questing adult *Ixodes ricinus* ticks from a natural focus in southeastern Germany. In this natural focus, several human cases with mild, mainly gastrointestinal, and constitutional symptoms, but no neurological symptoms, occurred in the nearby village. Ticks were crushed and nucleic acid extracted and tested as described (4). Sequencing was directly from the infected tick pool (E and NS2a genes) and from the primary isolation in Vero cell culture (whole genome).

Comparing the whole genome with those of the available TBEV whole genomes, the closest genetic relation was found to the TBEV strain Skrivanek (accession no. KJ922514.1) (5). The two strains differed in 13 amino acids, of which seven exchanges are conserved and six exchanges (two in NS1 and NS5 and one each in E and NS2b genes) are nonconserved and unique. The further genetic and phenotypic comparison with strain Skrivanek will be especially interesting, as it was isolated from a patient (Skrivanek) and therefore is pathogenic for humans. The TBEV strain MucAr HB171/11 might help give further insight in the pathogenetic potential of different TBEV strains.

### Accession number(s).

The whole-genome sequence of MucAR HB171/11 has been submitted in GenBank under the accession number KX268728.
